# From Collapse to Recovery: Thiamine Intervention in Cardiac Beriberi

**DOI:** 10.7759/cureus.54179

**Published:** 2024-02-14

**Authors:** Yazan Alamro, Khurram Arshad, Rabia Latif, Mohannad Al Akeel, Mohammad Ali Mozaffari

**Affiliations:** 1 Internal Medicine, Corewell Health Dearborn Hospital, Dearborn, USA; 2 Internal Medicine, McLaren Flint Hospital, Flint, USA; 3 Internal Medicine, East Tennessee State University Quillen College of Medicine, Johnson City, USA

**Keywords:** case report, cardio-circulatory collapse, pericardial effusion, thiamine deficiency, cardiac beriberi

## Abstract

This case report details the challenging presentation of a 68-year-old patient of cardio-circulatory collapse with pericardial effusion and recurrent pleural effusions. Hypotension did not respond to conventional intensive care measures. Despite comprehensive investigations, the underlying cause remained unidentified until intravenous thiamine replacement therapy was administered, restoring normotension and preventing pericardial or pleural effusion recurrence. The case underscores the importance of early recognition of thiamine deficiency in patients with pericardial and pleural effusions or critical illness, emphasizing the need to expand vigilance for thiamine deficiency and consider replacement therapy without a definitive diagnosis.

## Introduction

Thiamine deficiency, or beriberi, is a condition that results from a lack of thiamine (vitamin B1) in the body. The term beriberi comes from a Sinhalese word that means "extremely weak." There are two significant classifications of thiamine deficiency, which affect different body systems. The first is the cardiovascular system, known as wet beriberi, and the second is the nervous system, referred to as dry beriberi. Dry beriberi can present as peripheral neuropathy, Wernicke's encephalopathy, or Korsakoff syndrome, while wet beriberi typically presents as right-sided heart failure with elevated cardiac output [1]. However, identifying thiamine deficiency in complex clinical cases can be challenging, especially in patients without a history of alcohol abuse or overt malnutrition. This case report highlights a rare presentation of thiamine deficiency and emphasizes the importance of considering nutritional deficiencies in patients with unexplained cardiovascular symptoms.

## Case presentation

A 68-year-old male with a complex medical history presented with worsening dyspnea on exertion, progressive leg edema, and lower-extremity muscle weakness. Upon physical examination, the patient exhibited marked obesity, distant heart sounds, the presence of an S4 sound, and noticeable lower extremity swelling. 

The initial labs shown in Table 1 were remarkable for chronic anemia with a hemoglobin level of 8.6 g/dL (normal range: 13.5-17) and brain natriuretic peptide (BNP) of 286 pg/mL compared to a baseline of 55 pg/mL (normal range: 0-100). Severe hypoalbuminemia was observed at 1.6 g/dL, along with a low albumin level of 6 mg/dL (normal range: 18-44), suggesting nutritional deficiencies. Cardiac ischemia workup was unremarkable. 

**Table 1:** Initial investigation results

Investigations	Result	Normal range	Investigations	Result	Normal range
Sodium	137 mmol/L	136-144 mmol/L	Hemoglobin	8.6 g/dl	13.5-17 g/dl
potassium	4 mEq/L	3.5-5.5 mEq/L	White blood cell count^4^^4^	7 × 10^9^/L	4-11 × 10^9^/L
Chloride	105 mEq/L,	98-108 mEq/L,	Platelets	126 10^9^/L.	150-450 10^9^/L.
Blood urea nitrogen	14 mg/dL	7-22 mg/dL	B-type natriuretic peptide	286 pg/ml	0-100 pg/ml
Albumin	6 mg/dl	18-44 mg/dl	Mean corpuscular volume	977 fl	80-100 fl.

**Figure 1:**
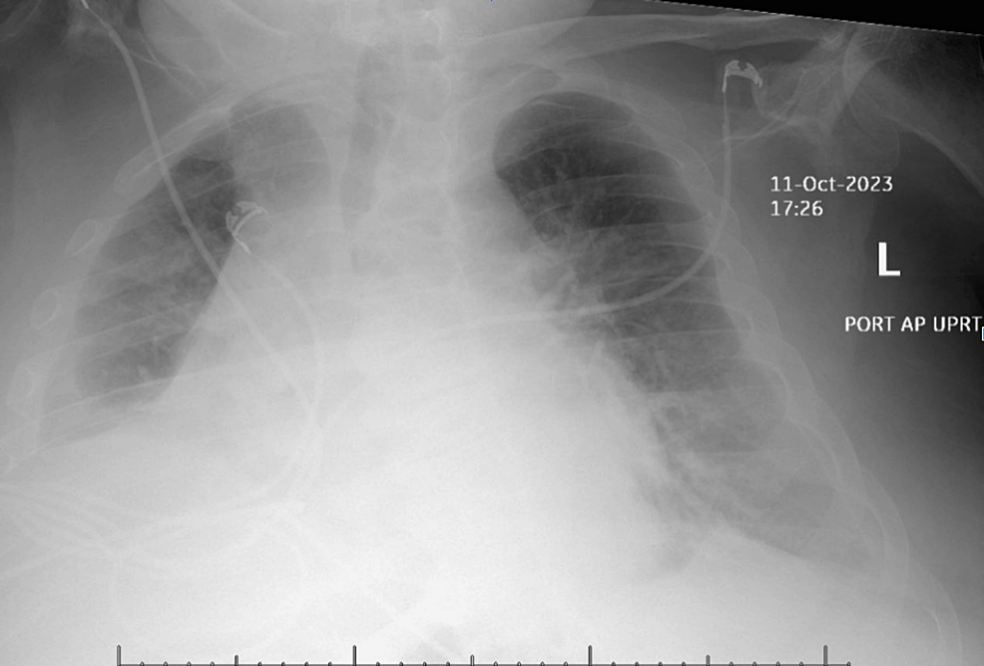
Cardiomegaly with moderate right-sided pleural effusion and adjacent compressive atelectasis. Small left pleural effusion.

**Figure 2:**
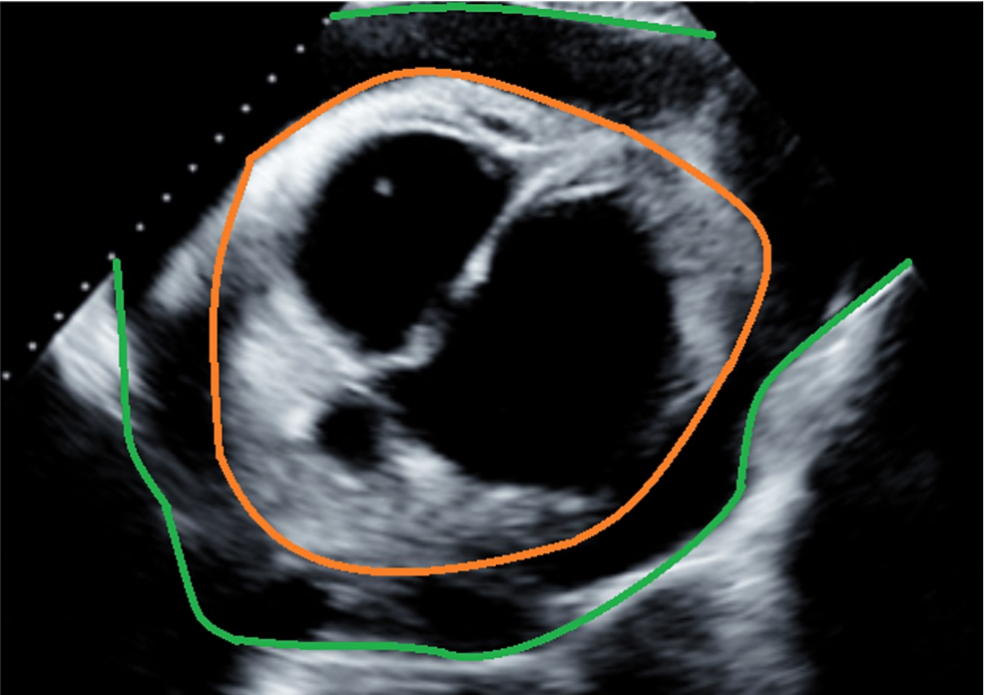
There is a large (>2 cm) pericardial effusion. The orange line is the epicardial border, and the green line is the pericardial border. The area between the two borders represents the pericardial space and effusion.

**Figure 3:**
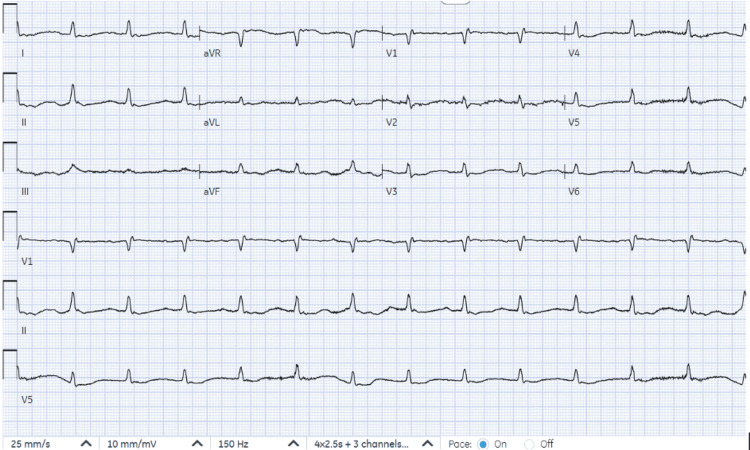
Atrial fibrillation, low-voltage QRS, and nonspecific ST and T wave abnormality.

Limited echocardiography was prompted by cardiomegaly observed on the initial portable chest X-ray (Figure 1). The echocardiogram revealed a pericardial effusion (Figure 2). The electrocardiogram (ECG) (Figure 3) exhibited low-voltage QRS complexes, atrial fibrillation, and non-specific T and ST changes. These findings prompted urgent pericardiocentesis. 

In the following week, the patient experienced hypotension and sinus tachycardia. Despite fluid resuscitation, the patient remained hemodynamically unstable. Vasopressor support and other adjuvant therapies were started, and a comprehensive workup was sent, which was unremarkable except for a borderline low serum thiamine level at 41 ug/L (normal range: 38-122) and persistent mild pericardial effusion on repeated limited echocardiogram. Analysis and cytology results from the pericardial and pleural fluids were unremarkable. 

**Figure 4:**
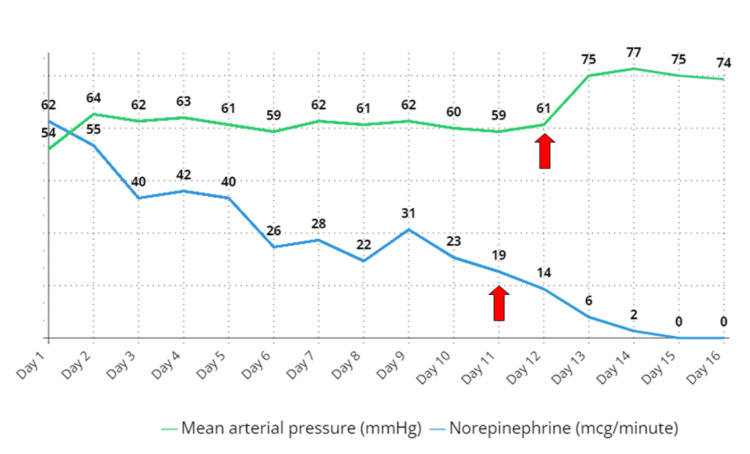
Chart representing the trend of mean arterial pressure (MAP) along with the vasopressor doses prior to and following thiamine infusion on day 11. The x-axis is the days, and the arrow points to dramatic changes in the mean arterial pressure (MAP) and vasopressor dose following thiamine infusion. In the chart, norepinephrine is shown in milliliters per minute. The red arrows represent the dramatic changes in the MAP and norepinephrine requirement.

Our patient was at risk for thiamine deficiency. Therefore, we administered intravenous thiamine replacement therapy at a dose of 100 mg three times per day. This resulted in a rapid improvement in hemodynamic stability. Over the next few days, the patient's vasopressor support was successfully discontinued, and both pericardial and pleural effusions did not recur in the follow-up period (Figure 4).

This case highlights the significance of considering wet beriberi as a potential cause of pericardial effusion and hemodynamic instability, especially in patients who are at risk for thiamine deficiency. The timely recognition and treatment of thiamine deficiency played a crucial role in resolving the patient's clinical presentation.

## Discussion

Thiamine deficiency can occur in cachectic patients and those with a calorie-rich yet nutritionally poor diet. Thiamine is an essential cofactor in enzymatic carbohydrate metabolism and energy production. Its deficiency disrupts adenosine triphosphate (ATP) production, which leads to adenosine accumulation. This accumulation causes direct vasomotor depression and reduced systemic vascular resistance. The decreased vascular resistance triggers fluid retention through the renin-angiotensin-aldosterone system, worsening peripheral edema, especially in dependent areas, such as the legs [2]. As thiamine deficiency progresses, the heart, which is already compromised in energy production, faces an increased workload to meet organ demands. This eventually leads to hypotension and cardiovascular collapse unless thiamine is provided [3].

In our patient, we suspected wet beriberi due to several risk factors, malnutrition, and cardiovascular collapse. We initiated immediate thiamine replacement therapy before receiving laboratory results because diagnosing thiamine deficiency through blood tests can be limited. Plasma thiamine levels, often influenced by recent caloric intake, may not accurately reflect deficiency, especially in nonfasting states. Moreover, anemia can affect erythrocyte transketolase activity (ETKA), another diagnostic indicator. In this context, the diagnosis was based on an unexplained cardiomyopathy presentation, poor nutrition history, therapeutic response to thiamine, and the exclusion of alternative heart disease etiologies [4].

The recommended treatment for thiamine deficiency varies depending on the severity of the case, but it usually involves intravenous supplementation, especially for critically ill patients. The loading dose can range from 100 mg to 500 mg of thiamine given through an injection, followed by a daily oral regimen of 25 to 100 mg. Studies have suggested that up to 30% of patients with acute heart failure may have thiamine deficiency and that thiamine supplementation can improve ejection fraction by up to 10%. Although thiamine deficiency is recognized as a cause of cardiomyopathy, the American Heart Association/American College of Cardiology guidelines do not provide specific recommendations for thiamine deficiency evaluation [5-8].

This case underscores the importance of clinical acumen in diagnosing thiamine deficiency, especially when laboratory tests may be inconclusive. Timely initiation of thiamine replacement proved crucial in resolving the patient's cardiovascular collapse and highlights the need for increased awareness of thiamine deficiency in diverse clinical presentations.

## Conclusions

The passage underscores the underdiagnosis of thiamine deficiency, such as beriberi, within the US healthcare system, highlighting its severe yet treatable nature. It emphasizes the significance of early detection and swift treatment to reverse associated cardiovascular issues, particularly in cases of rapidly developing cardiomyopathy among populations susceptible to alcoholism and malnutrition. Despite diagnostic challenges, the recommendation is to assess patients with unexplained cardiomyopathy symptoms for thiamine deficiency. The narrative stresses the critical role of early suspicion and immediate treatment, advocating for heightened clinician awareness and a proactive approach to assessing and treating thiamine deficiency, ultimately leading to improved patient outcomes.
